# Supplementary material for: Learning dynamically regulates stimulus discrimination of ventral striatal D1 receptor expressing neurons
Source: Sci Adv. 2026 Jul 15;12(29):eaee3529. doi: 10.1126/sciadv.aee3529 (PMC13371909; doi:10.1126/sciadv.aee3529)
Supplement: Supplementary file 1 — Figs. S1 to S7 [file sciadv.aee3529_sm.pdf]

Science Advances
AAAS

Supplementary Materials for

Learning dynamically regulates stimulus discrimination of ventral striatal D1 receptor expressing neurons

Tierney B. Daw *et al.*

Corresponding author: Sotiris C. Masmanidis, smasmanidis@ucla.edu

Sci. Adv. **12**, eaee3529 (2026)
DOI: 10.1126/sciadv.aee3529

This PDF file includes:

Figs. S1 to S7

SUPPLEMENTARY MATERIALS

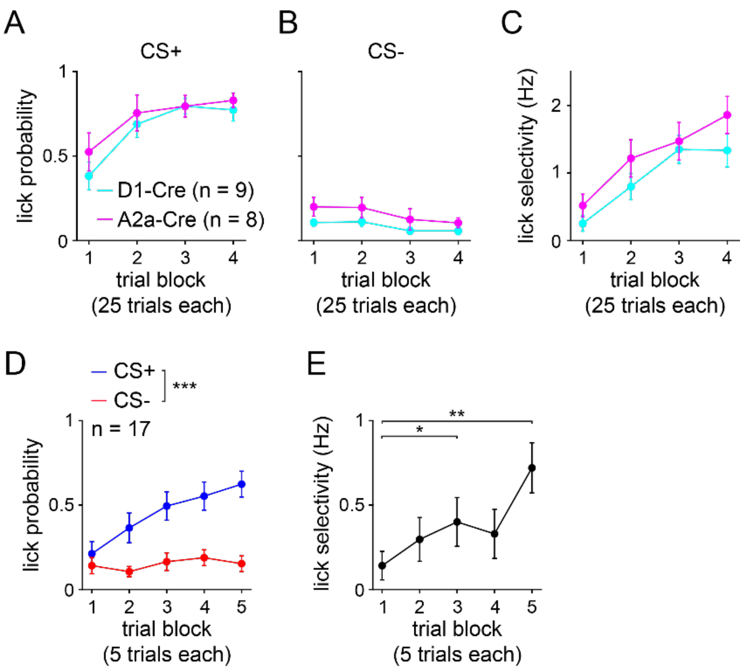

Fig. S1. Behavior of D1-Cre and A2a-Cre mice and initial responses to cues. (**A**) CS+ lick probability plotted separately for D1-Cre and A2a-Cre mice (*n* = 9 D1-Cre and 8 A2a-Cre mice; two-way RM ANOVA; block effect: $F_{3,45}$ = 15, P < 0.0001; group effect: $F_{1,15}$ = 0.7, P = 0.43). (**B**) Same as A, but for CS- (two-way RM ANOVA; block effect: $F_{3,45}$ = 4, P = 0.022; cell type effect: $F_{1,15}$ = 3, P = 0.11). (**C**) Same as A, but for lick selectivity (two-way RM ANOVA; block effect: $F_{3,45}$ = 21, P < 0.0001; cell type effect: $F_{1,15}$ = 1.8, P = 0.19). (**D**) CS+ and CS- lick probabilities across the initial five 5-trial blocks (*n* = 17 mice; two-way RM ANOVA; block effect: $F_{4,128}$ = 9, P < 0.0001; cue effect: $F_{1,32}$ = 16, P = 0.0004; multiple comparisons for CS+ versus CS- on blocks 1 (P = 0.95), 2 (P = 0.030), 3 (P = 0.0025), 4 (P = 0.0006), and 5 (P < 0.0001)). (**E**) Lick selectivity versus trial block (one-way RM ANOVA; block effect: $F_{3,42}$ = 5, P = 0.0057; multiple comparisons for blocks 1 versus 2 (P = 0.37), 3 (P = 0.025), 4 (P = 0.51), and 5 (P = 0.0017)). A-E depict mean ± SEM. * P < 0.05, ** P < 0.01, *** P < 0.001.

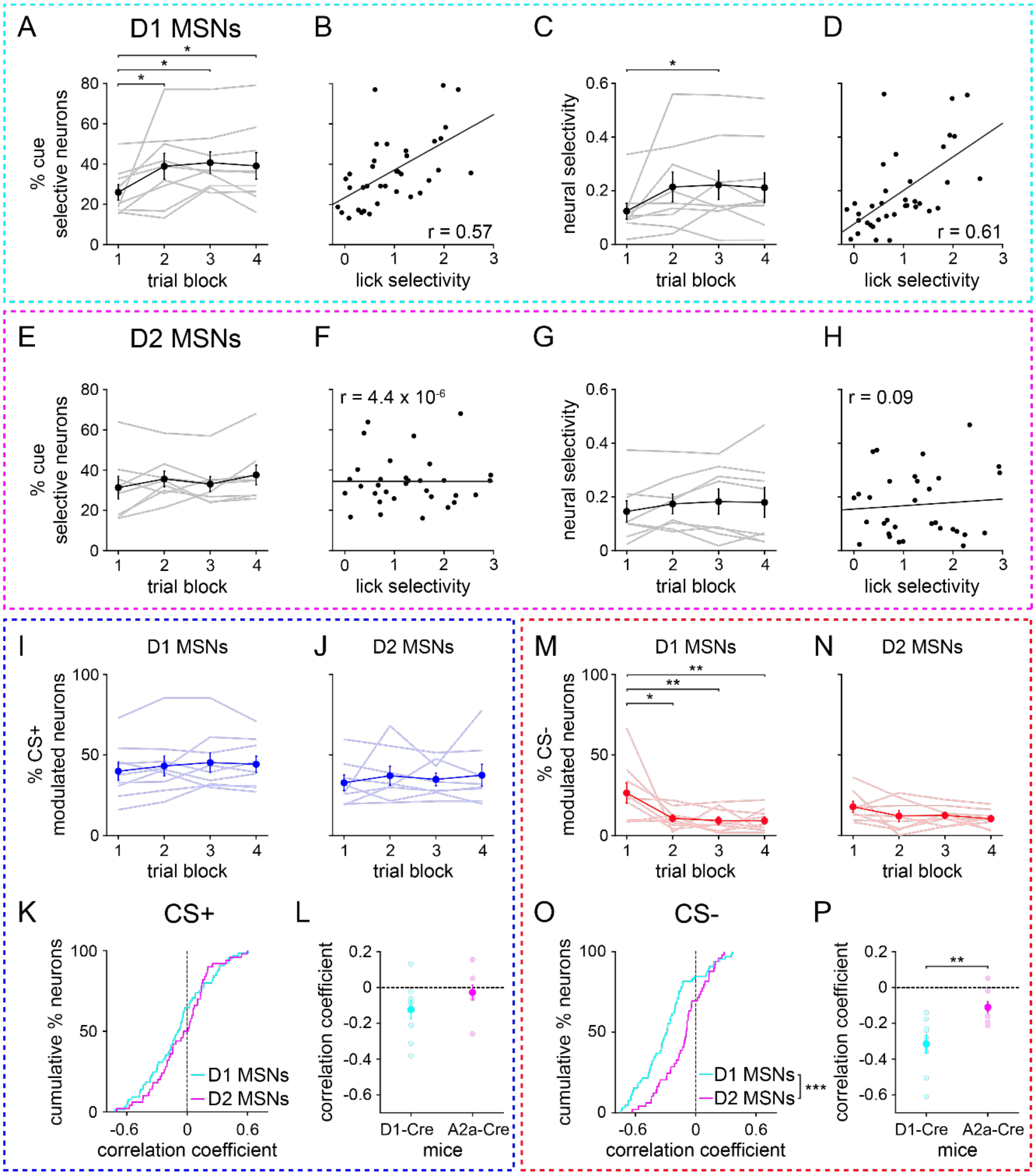

Fig. S2. More stringent opto-tagging criteria replicate the main results. (**A**) Percentage of D1 MSNs selectively responsive to CS+ or CS- versus trial block (*n* = 9 D1-Cre mice; one-way RM ANOVA; block effect: $F_{3,24}$ = 4, P = 0.018; multiple comparisons for blocks 1 versus 2 (P = 0.033), 3 (P = 0.014), and 4 (P = 0.030)). (**B**) Percentage of cue-selective D1 MSNs versus lick selectivity (*n* = 36 (9 mice and 4 trial blocks); Pearson correlation coefficient; r = 0.57, P = 0.0003). (**C**) D1 MSN selectivity versus trial block (one-way RM ANOVA; block effect: $F_{3,24}$ = 3, P = 0.041; multiple comparisons for blocks 1 versus 2 (P = 0.053), 3 (P = 0.034), and 4 (P = 0.062)). (**D**) D1 MSN selectivity versus lick selectivity (Pearson correlation coefficient; r = 0.61, P < 0.0001). (**E-H**) Same as A-D, but for D2 MSNs. (**E**) (*n* = 8 A2a-Cre mice; one-way RM ANOVA; block effect: $F_{3,21}$ = 1.7, P = 0.20). (**F**) (*n* = 32 (8 mice and 4 trial blocks); Pearson correlation coefficient; r = 4.4×10^{-6}, P > 0.99). (**G**) (one-way RM ANOVA; block effect: $F_{3,21}$ = 1.1, P = 0.36). (**H**) (Pearson

correlation coefficient; $r = 0.09$, $P = 0.64$). (**I**) Percentage of CS+ modulated D1 MSNs versus trial block (one-way RM ANOVA; block effect: $F_{3,24} = 1.1$, $P = 0.37$). (**J**) Same as I, but for D2 MSNs (one-way RM ANOVA; block effect: $F_{3,21} = 0.4$, $P = 0.78$). (**K**) Cumulative percentage of D1 and D2 MSNs versus correlation coefficient between CS+ firing rate and trial number (first 25 trials) ($n = 66$ D1 and 50 D2 MSNs; two-sample Kolmogorov-Smirnov test; $P = 0.47$). (**L**) Same as K, but plotted per animal ($n = 9$ D1-Cre and 8 A2a-Cre mice; two-sample t-test; $P = 0.18$). (**M-P**) Same as I-L, but for CS-. (**M**) (one-way RM ANOVA; block effect: $F_{3,24} = 6$, $P = 0.0039$; multiple comparisons for blocks 1 versus 2 ($P = 0.010$), 3 ($P = 0.0051$), and 4 ($P = 0.0050$)). (**N**) (one-way RM ANOVA; block effect: $F_{3,21} = 1.7$, $P = 0.20$). (**O**) (two-sample Kolmogorov-Smirnov test; $P = 0.0001$). (**P**) (two-sample t-test; $P = 0.0048$). All panels depict data using the 3 ms maximum latency criterion for opto-tagging. Dark colors in A, C, E, G, I-J, L-N, and P depict mean ± SEM; light colors depict individual animal data. * $P < 0.05$, ** $P < 0.01$.

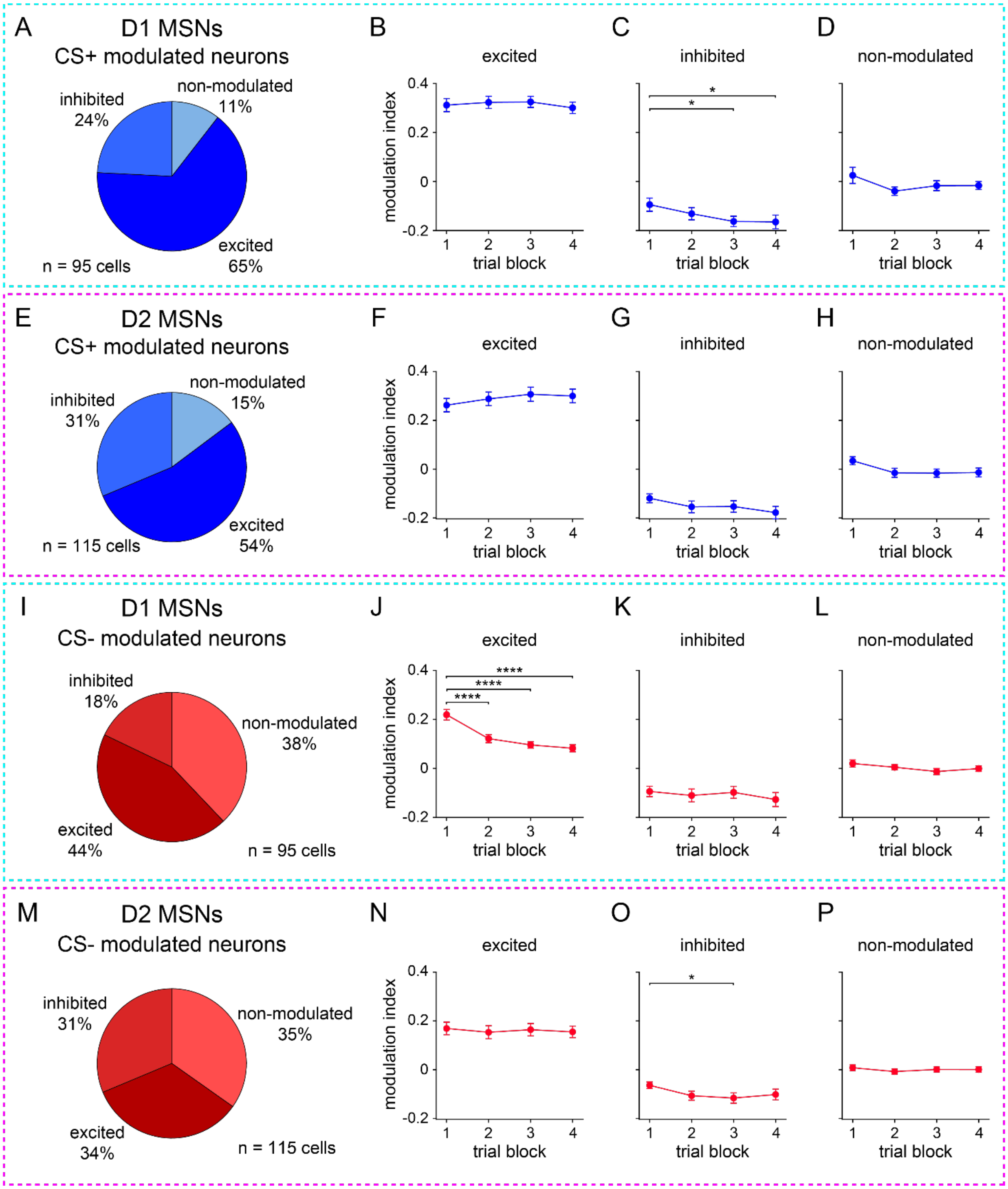

Fig. S3. D1 and D2 MSNs exhibit mixed responses to predictive cues. (**A**) Proportion of D1 MSNs that were excited, inhibited, or non-modulated by CS+. (**B**) Modulation index versus trial block for D1 MSNs excited by CS+ (n = 62 D1 MSNs; one-way RM ANOVA; block effect: $F_{3,183}$ = 0.9, P = 0.44). (**C**) Same as B, but for D1 MSNs inhibited by CS+ (n = 23 D1 MSNs; one-way RM ANOVA; block effect: $F_{3,66}$ = 3, P = 0.031; multiple comparisons for blocks 1 versus 2 (P = 0.37), 3 (P = 0.032), and 4 (P = 0.026)). (**D**) Same as B, but for D1 MSNs non-modulated by CS+ (n = 10 D1 MSNs; one-way RM ANOVA; block effect: $F_{3,27}$ = 1.5, P = 0.23). (**E-H**) Same as A-D, but for D2 MSNs. (**F**) (n = 62 D2 MSNs; one-way RM ANOVA; block effect: $F_{3,183}$ = 2, P = 0.11). (**G**)

(*n* = 36 D2 MSNs; one-way RM ANOVA; block effect: $F_{3,105}$ = 2, *P* = 0.086). (**H**) (*n* = 17 D2 MSNs; one-way RM ANOVA; block effect: $F_{3,48}$ = 2, *P* = 0.11). (**I-L**) Same as A-D, but for CS-. (**J**) (*n* = 42 D1 MSNs; one-way RM ANOVA; block effect: $F_{3,123}$ = 22, *P* < 0.0001; multiple comparisons for blocks 1 versus 2-4 (*P* < 0.0001)). (**K**) (*n* = 17 D1 MSNs; one-way RM ANOVA; block effect: $F_{3,48}$ = 0.7, *P* = 0.55). (**L**) (*n* = 36 D1 MSNs; one-way RM ANOVA; block effect: $F_{3,105}$ = 1.3, *P* = 0.27). (**M-P**) Same as E-H, but for CS-. (**N**) (*n* = 39 D2 MSNs; one-way RM ANOVA; block effect: $F_{3,114}$ = 0.2, *P* = 0.87). (**O**) (*n* = 36 D2 MSNs; one-way RM ANOVA; block effect: $F_{3,105}$ = 3, *P* = 0.048; multiple comparisons for blocks 1 versus 2 (*P* = 0.082), 3 (*P* = 0.025), and 4 (*P* = 0.14)). (**P**) (*n* = 40 D2 MSNs; one-way RM ANOVA; block effect: $F_{3,117}$ = 0.3, *P* = 0.80). B-D, F-H, J-L, and N-P depict mean ± SEM. * *P* < 0.05, **** *P* < 0.0001.

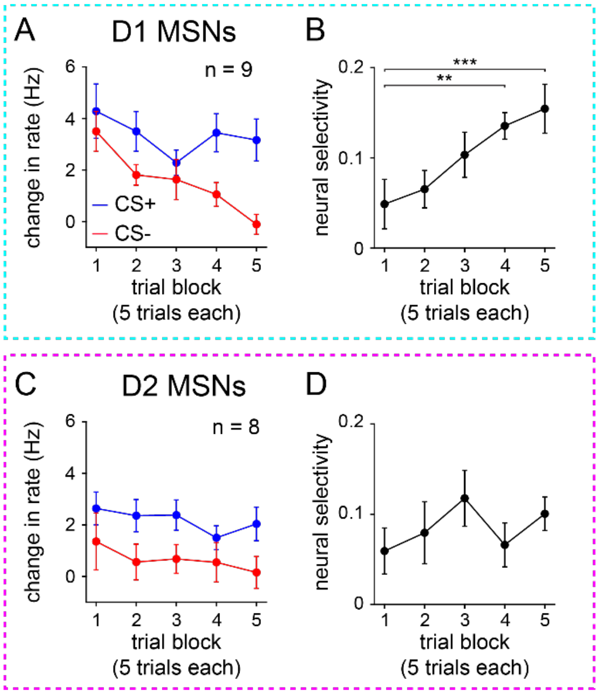

Fig. S4. Changes in D1 MSN activity are evident early in learning. (**A**) D1 MSN baseline-subtracted firing rate versus trial block (n = 9 D1-Cre mice; two-way RM ANOVA; block effect: $F_{4,64}$ = 9, P < 0.0001; cue effect: $F_{1,16}$ = 4, P = 0.051). (**B**) D1 MSN selectivity versus trial block (one-way RM ANOVA; block effect: $F_{4,32}$ = 6, P = 0.0009; multiple comparisons for blocks 1 versus 2 (P = 0.92), 3 (P = 0.13), 4 (P = 0.0068), and 5 (P = 0.0009)). (**C**) Same as A, but for D2 MSNs (n = 8 A2a-Cre mice; two-way RM ANOVA; block effect: $F_{4,56}$ = 1.3, P = 0.26; cue effect: $F_{1,14}$ = 4, P = 0.067). (**D**) Same as B, but for D2 MSNs (one-way RM ANOVA; block effect: $F_{4,28}$ = 1.5, P = 0.23). A-D depict mean ± SEM. ** P < 0.01, *** P < 0.001.

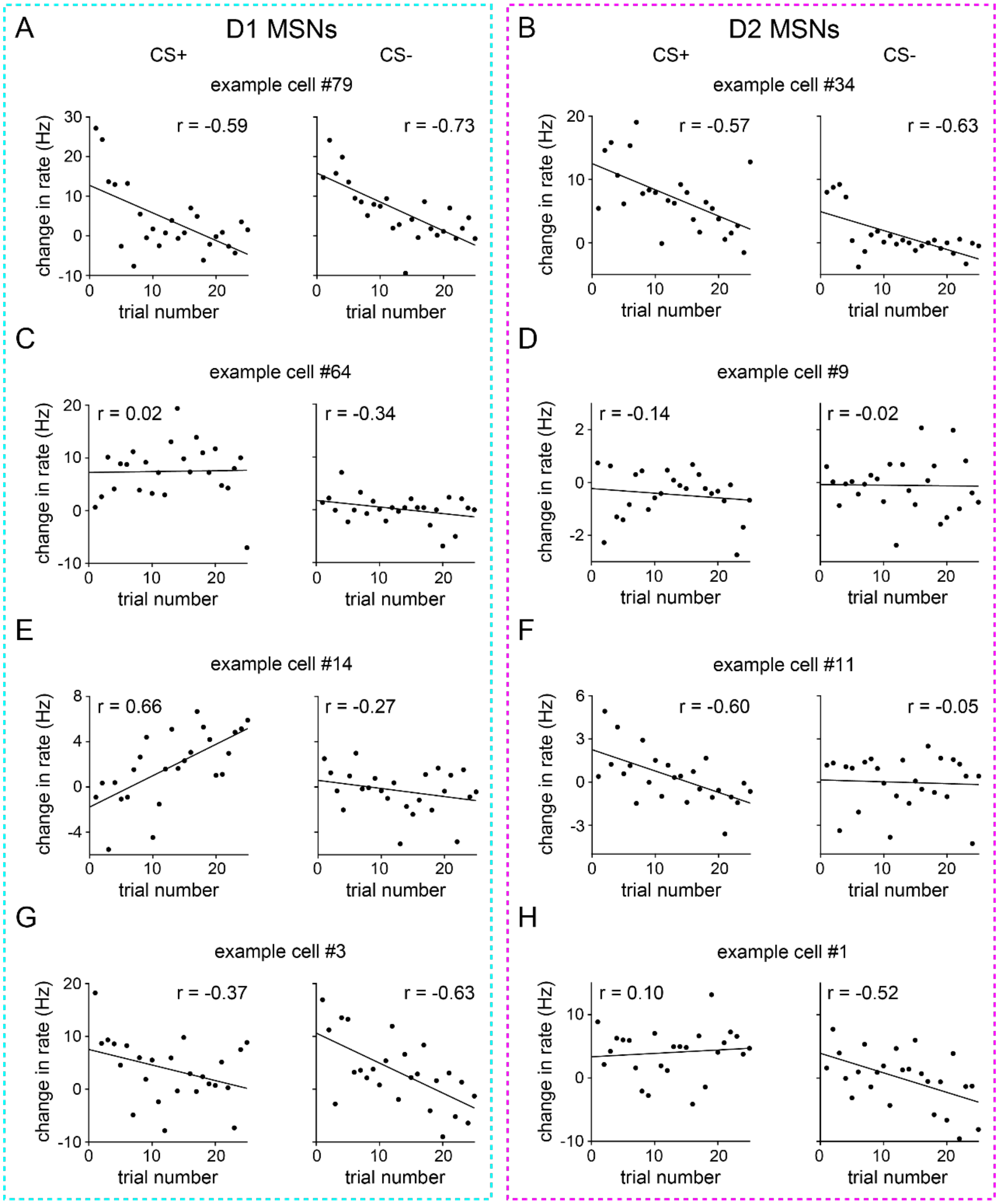

Fig. S5. MSNs exhibit heterogenous changes in firing rate early in learning. (**A**) D1 MSN with significantly decreasing firing rates to both CS+ and CS-. CS+ (n = 25 trials; Pearson correlation coefficient; r = -0.59, P = 0.0018) and CS- (Pearson correlation coefficient; r = -0.73, P < 0.0001). (**B**) Same as A, but for D2 MSNs. CS+ (Pearson correlation coefficient; r = -0.57, P = 0.0027) and CS- (Pearson correlation coefficient; r = -0.63, P = 0.0007). (**C**) D1 MSN with no significant changes in firing rates to either CS+ or CS-. CS+ (Pearson correlation coefficient; r = 0.02, P = 0.91) and CS- (Pearson correlation coefficient; r = -0.34, P = 0.098). (**D**) Same as C, but for D2 MSNs. CS+ (Pearson correlation coefficient; r = -0.14, P = 0.50) and CS- (Pearson correlation coefficient; r = -0.02, P = 0.93). (**E**) D1 MSN with significant change in firing rates to CS+, but not CS-. CS+ (Pearson correlation coefficient; r = 0.66, P = 0.0004) and CS- (Pearson

correlation coefficient; *r* = -0.27, *P* = 0.19). (**F**) Same as E, but for D2 MSNs. CS+ (Pearson correlation coefficient; *r* = -0.60, *P* = 0.0014) and CS- (Pearson correlation coefficient; *r* = -0.05, *P* = 0.81). (**G**) D1 MSN with significant change in firing rates to CS-, but not CS+. CS+ (Pearson correlation coefficient; *r* = -0.37, *P* = 0.072) and CS- (Pearson correlation coefficient; *r* = -0.63, *P* = 0.0007). (**H**) Same as G, but for D2 MSNs. CS+ (Pearson correlation coefficient; *r* = 0.10, *P* = 0.63) and CS- (Pearson correlation coefficient; *r* = -0.52, *P* = 0.0078). All panels depict data from the first 25 trials of each cue.

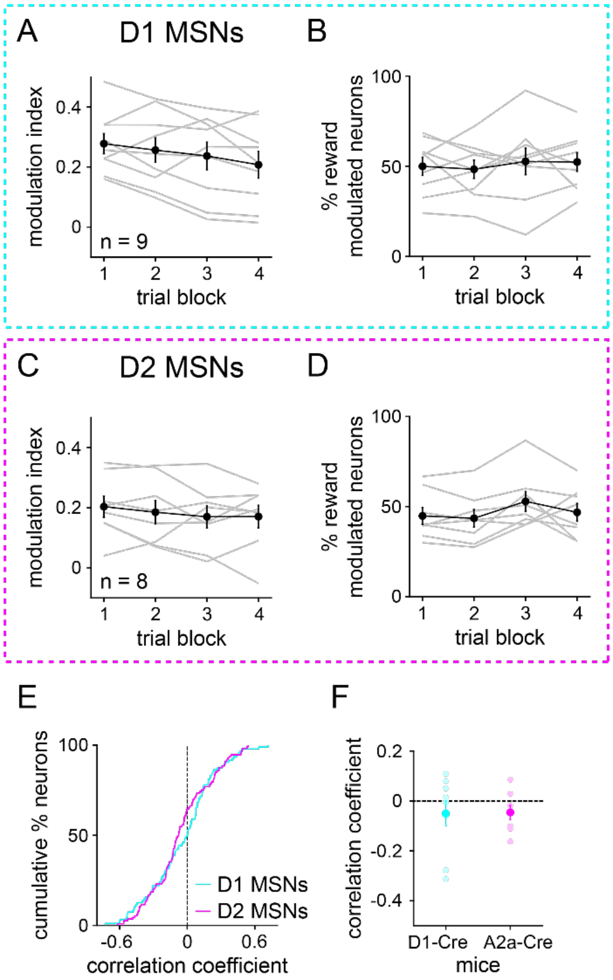

Fig. S6. MSNs exhibit fixed average reward activity during learning. (**A**) D1 MSN modulation index versus trial block (*n* = 9 D1-Cre mice; one-way RM ANOVA; block effect: $F_{3,24}$ = 3, *P* = 0.067). (**B**) Percentage of reward modulated D1 MSNs versus trial block (one-way RM ANOVA; block effect: $F_{3,24}$ = 0.4, *P* = 0.78). (**C**) Same as A, but for D2 MSNs (*n* = 8 A2a-Cre mice; one-way RM ANOVA; block effect: $F_{3,21}$ = 0.6, *P* = 0.60). (**D**) Same as B, but for D2 MSNs (one-way RM ANOVA; block effect: $F_{3,21}$ = 2, *P* = 0.095). (**E**) Cumulative percentage of D1 and D2 MSNs versus correlation coefficient between reward firing rate and trial number (first 25 trials) (*n* = 95 D1 and 115 D2 MSNs; two-sample Kolmogorov-Smirnov test; *P* = 0.17). (**F**) Same as E, but plotted per animal (*n* = 9 D1-Cre and 8 A2a-Cre mice; two-sample t-test; *P* = 0.94). Dark colors in A-D and F depict mean ± SEM; light colors depict individual animal data.

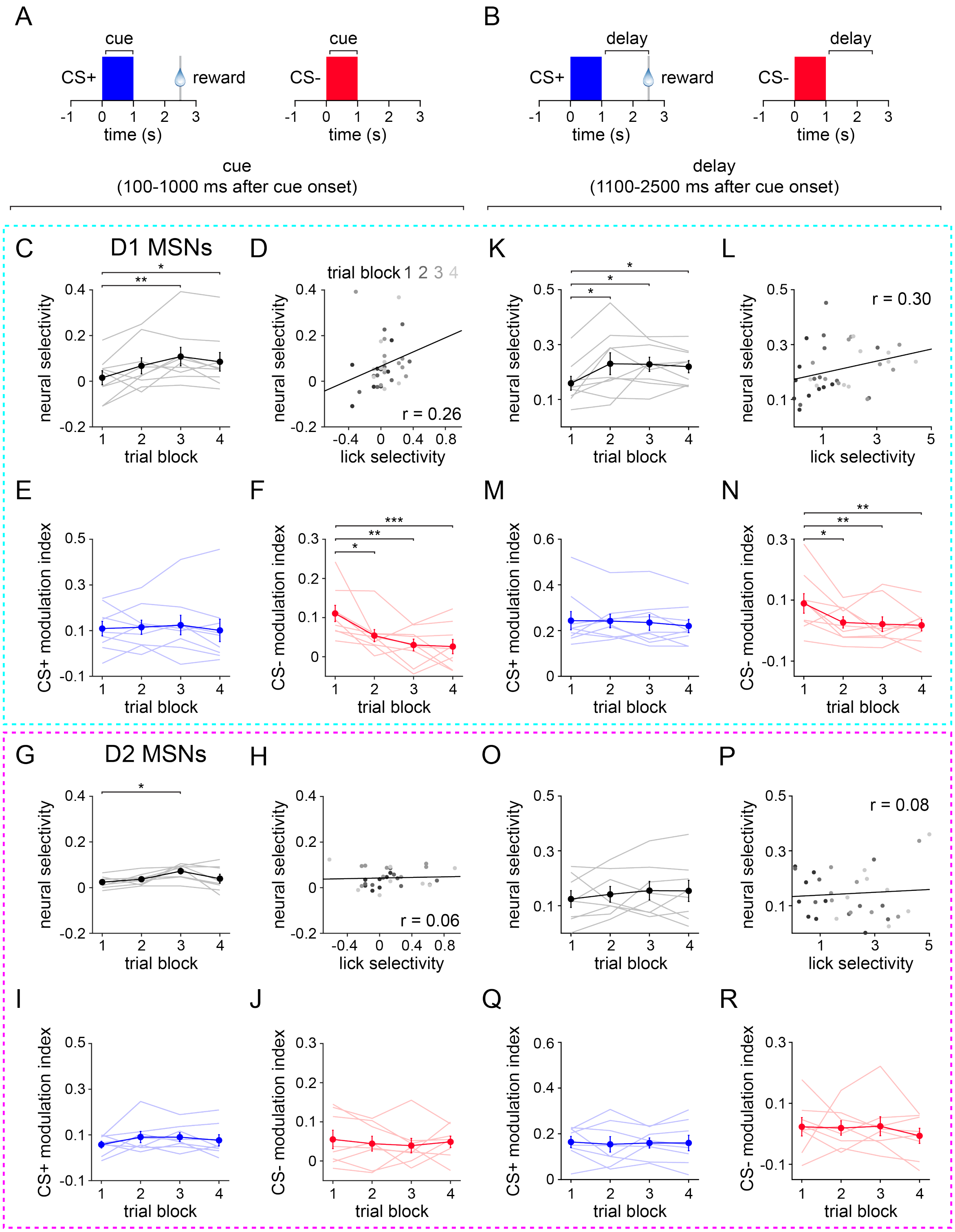

Fig. S7. Cue and delay period analyses replicate the main results. (**A-B**) Schematic of the cue and delay periods. (**C-F**) D1 MSN analysis during the cue period (100-1000 ms after cue onset). (**C**) D1 MSN selectivity versus trial block (one-way RM ANOVA; block effect: $F_{3,24} = 6$, $P = 0.0038$; multiple comparisons for blocks 1 versus 2 ($P = 0.083$), 3 ($P = 0.0014$), and 4 ($P = 0.016$)). (**D**) D1 MSN selectivity versus lick selectivity (Pearson correlation coefficient; $r = 0.26$, $P = 0.12$). Points are colored by trial block. (**E**) D1 MSN CS+ modulation index versus trial block (one-way

RM ANOVA; block effect: $F_{3,24}$ = 0.3, *P* = 0.82). (**F**) D1 MSN CS- modulation index versus trial block (one-way RM ANOVA; block effect: $F_{3,24}$ = 8, *P* = 0.0008; multiple comparisons for blocks 1 versus 2 (*P* = 0.022), 3 (*P* = 0.0012), and 4 (*P* = 0.0007)). (**G-J**) Same as C-F, but for D2 MSNs. (**G**) (one-way RM ANOVA; block effect: $F_{3,21}$ = 4, *P* = 0.026; multiple comparisons for blocks 1 versus 2 (*P* = 0.73), 3 (*P* = 0.011), and 4 (*P* = 0.62)). (**H**) (Pearson correlation coefficient; *r* = 0.06, *P* = 0.75). (**I**) (one-way RM ANOVA; block effect: $F_{3,21}$ = 1.5, *P* = 0.25). (**J**) (one-way RM ANOVA; block effect: $F_{3,21}$ = 0.3, *P* = 0.80). (**K-N**) Same as C-F, but for the delay period (1100-2500 ms after cue onset). (**K**) (one-way RM ANOVA; block effect: $F_{3,24}$ = 4, *P* = 0.019; multiple comparisons for blocks 1 versus 2 (*P* = 0.017), 3 (*P* = 0.021), and 4 (*P* = 0.046)). (**L**) (Pearson correlation coefficient; *r* = 0.30, *P* = 0.075). (**M**) (one-way RM ANOVA; block effect: $F_{3,24}$ = 0.5, *P* = 0.71). (**N**) (one-way RM ANOVA; block effect: $F_{3,24}$ = 6, *P* = 0.0048; multiple comparisons for blocks 1 versus 2 (*P* = 0.014), 3 (*P* = 0.0073), and 4 (*P* = 0.0048)). (**O-R**) Same as K-N, but for D2 MSNs. (**O**) (one-way RM ANOVA; block effect: $F_{3,21}$ = 0.6, *P* = 0.62). (**P**) (Pearson correlation coefficient; *r* = 0.08, *P* = 0.67). (**Q**) (one-way RM ANOVA; block effect: $F_{3,21}$ = 0.1, *P* = 0.98). (**R**) (one-way RM ANOVA; block effect: $F_{3,21}$ = 0.5, *P* = 0.71). Dark colors in C, E-G, I-K, M-O, and Q-R depict mean ± SEM; light colors depict individual animal data. * *P* < 0.05, ** *P* < 0.01, *** *P* < 0.001.
